# Notoginsenoside R1 (NGR1) regulates the AGE-RAGE signaling pathway by inhibiting RUNX2 expression to accelerate ferroptosis in breast cancer cells

**DOI:** 10.18632/aging.205940

**Published:** 2024-06-14

**Authors:** Wenxin Li, Yan Guo, Zhuangyu Xu, Fubo Li, Yi Dong, Fan Xu

**Affiliations:** 1 Departments of Oncology, Affiliated Hospital of Chengde Medical University, Chengde, China

**Keywords:** Notoginsenoside R1, BRCA, RUNX2, AGE-RAGE, ferroptosis

## Abstract

Ferroptosis is a new way of cell death, and stimulating the process of cell ferroptosis is a new strategy to treat breast cancer. NGR1 has good anti-cancer activity and is able to slow the progression of breast cancer. However, NGR1 has not been reported in the field related to ferroptosis. By searching the online database for potential targets of NGR1 and the breast cancer disease database, among 11 intersecting genes we focused on Runt-related transcription factor 2 (RUNX2), which is highly expressed in breast cancer, and KEGG pathway enrichment showed that the intersecting genes were mainly enriched in the AGE (advanced glycosylation end products)-RAGE (receptor of AGEs) signaling pathway. After that, we constructed overexpression and down-regulation breast cancer cell lines of RUNX2 *in vitro*, and tested whether NGR1 treatment induced ferroptosis in breast cancer cells by regulating RUNX2 to inhibit the AGE-RAGE signaling pathway through phenotyping experiments of ferroptosis, Western blot experiments, QPCR experiments, and electron microscopy observation. The results showed that NGR1 was able to inhibit the expression level of RUNX2 and suppress the AGE/PAGE signaling pathway in breast cancer cells. NGR1 was also able to promote the accumulation of Fe^2+^ and oxidative damage in breast cancer cells by regulating RUNX2 and then down-regulating the expression level of GPX4, FIH1 and up-regulating the expression level of ferroptosis-related proteins such as COX2, ACSL4, PTGS2 and NOX1, which eventually led to the ferroptosis of breast cancer cells.

## INTRODUCTION

Breast cancer is the leading cause of death among women, and according to the latest statistics on breast cancer from the American Cancer Society, the incidence of breast cancer has increased annually over the past 40 years, with a 0.5% annual increase in incidence over the past decade (2010-2019), and there is a correlation between the increase in incidence of breast cancer and geography, estrogen levels, lifestyle habits, and dietary habits. Although the incidence of breast cancer continues to be high, the mortality rate has decreased by about 43% over the last 40 years [1, 2]. This is closely related to the advancement of early screening technology as well as the development of anticancer drugs and the improvement of people’s living habits. Although there has been a downward trend in the mortality rate of breast cancer, the poor prognosis of breast cancer still exists [3]. It has been reported that more than 90% of breast cancer patients’ deaths are related to metastasis [4] Metastasis of breast cancer is a complex and diverse process. We need to explore the differences in the pathogenesis of different types of breast cancer and its relationship with environmental factors. We need to find more effective therapeutic targets to provide more possibilities for the prevention, diagnosis and treatment of breast cancer.

Ferroptosis (iron death) was first proposed by Dr. Brent R. Stockwell of Columbia University in 2012, at the time it was seen as a new type of cell death that might be important for cell death [5]. It has been suggested that in breast cancer ferroptosis may determine tumor progression by influencing tumor cell survival and death. In addition, by regulating lipid metabolism and iron ion metabolism in tumor cells, ferroptosis may affect the migration and invasive ability of breast cancer cells [6] The study of the mechanism of ferroptosis in breast cancer metastasis provides new perspectives and therapeutic targets. Active saponins isolated from natural plants are highly promising anticancer active ingredients, and it has been reported that Astragaloside IV [7] Ginsenoside Rg1 [8], Timosaponin AIII [9], Saikosaponin A [10] and other saponins are able to slow down tumor progression by inducing the accumulation of iron ions, enhancing lipid peroxidation, and regulating ferroptosis. It is suggested that our saponin analogs are closely related to intracellular ferroptosis.

NGR1 is one of the medicinal components of ginseng Panax ginseng. NGR1 has a wealth of pharmacological effects, including the ability to improve cardiac repair after myocardial infarction [11] NGR1 can improve retinopathy in diabetic patients [12] and promoting post-stroke angiogenesis, etc. [13]. In addition, NGR1 has been shown to have a beneficial effect on a variety of cancers. In addition, NGR1 has an inhibitory effect on various cancer cells. NGR1 can inhibit the invasion and metastasis of colorectal cancer cells by inhibiting the expression of MMP9 [14]. NGR1 can inhibit the invasion and metastasis of colorectal cancer cells by inhibiting MMP9 expression. In breast cancer, NGR1 can regulate the expression of CCND2 and YBX3 to inhibit breast cancer angiogenesis and slow down the progression of breast cancer [15], but NGR1’s ferroptosis-associated neighborhood has not been reported. Based on the online database, we analyzed the novel target genes and signaling pathways of NGR1 and breast cancer disease, and explored whether NGR1 could regulate ferroptosis in breast cancer and thus affect the progression of breast cancer cells, with the aim of providing new drug targets and therapeutic options for breast cancer patients.

## RESULTS

### NGR1 cross-talk genes in breast cancer are mainly enriched in the AGE-RACE signaling pathway

**Figure 1:**
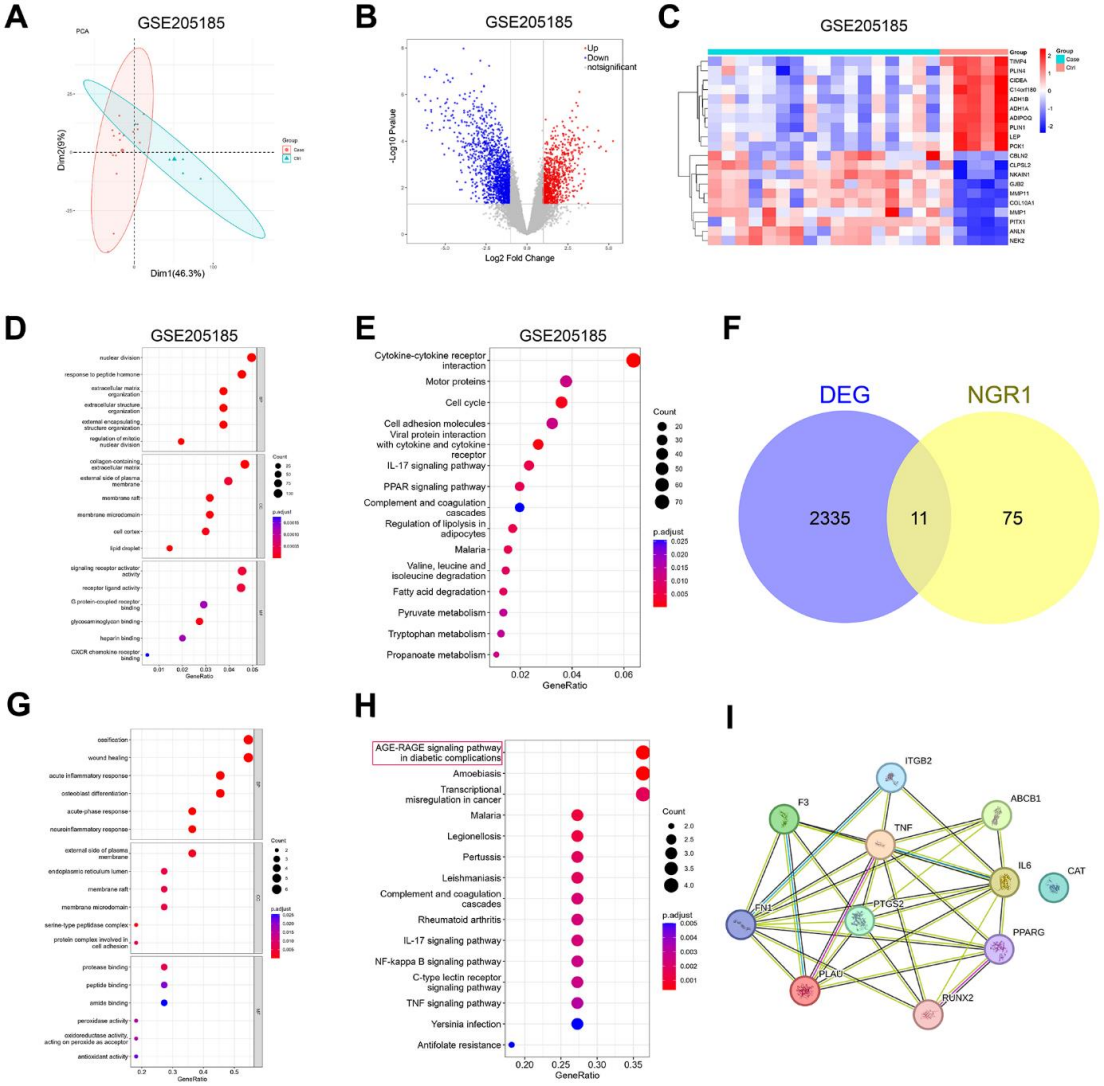
**Prediction of potential target genes and signaling pathways of NGR1 in breast cancer.** (**A**) The GSE205185 dataset was downloaded, and the R package “FactoMineR” and “factoextra” were used to perform principal component analysis on the two sets of samples. (**B**) Volcano map of differentially expressed genes in the GSE205185 dataset (**C**) Heat map of differentially expressed genes clustering in the GSE205185 dataset graph. (**D**) GSE205185 differential gene GO function enrichment analysis. (**E**) GSE205185 differential gene KEGG function enrichment analysis. (**F**) CTD database download NGR1 target gene and GSE205185 dataset plotting Wayne’s diagram. (**G**) Intersecting gene GO function enrichment analysis. (**H**) Intersecting gene KEGG functional enrichment analysis. (**I**) Intersecting genes PPI network diagrams.

Principal component analysis (PCA) was performed on the GSE205185 dataset, and the results were shown in Figure 1. PCA results showed that the two groups had different clusters. In the GSE205185 dataset, we plotted volcano map (Figure 1B), clustering heat map (Figure 1C), GO functional enrichment analysis (Figure 1D), KEGG functional enrichment analysis (Figure 1E), did not look for the results we were interested in, and after that, the online database prediction of potential targets of NGR1 with the breast cancer disease database Wehn diagrams showed that there were a total of 11 intersecting genes (Figure 1F). GO functional enrichment analysis showed that differential genes were mainly enriched in ossification, wound healing and acute inflammatory response. We found no information of interest here (Figure 1G). KEGG pathway enrichment analysis showed that the intersecting genes were mainly enriched in the AGE-RAGE signaling pathway (Figure 1H). So our follow-up experiments focused on AGE-RAGE signaling pathway. We then analyzed the differential genes through PPI protein interaction network. The results showed that all the other proteins except CAT proteins had partial interactions (Figure 1I).

### Screening of potential therapeutic targets for NGR1

**Figure 2:**
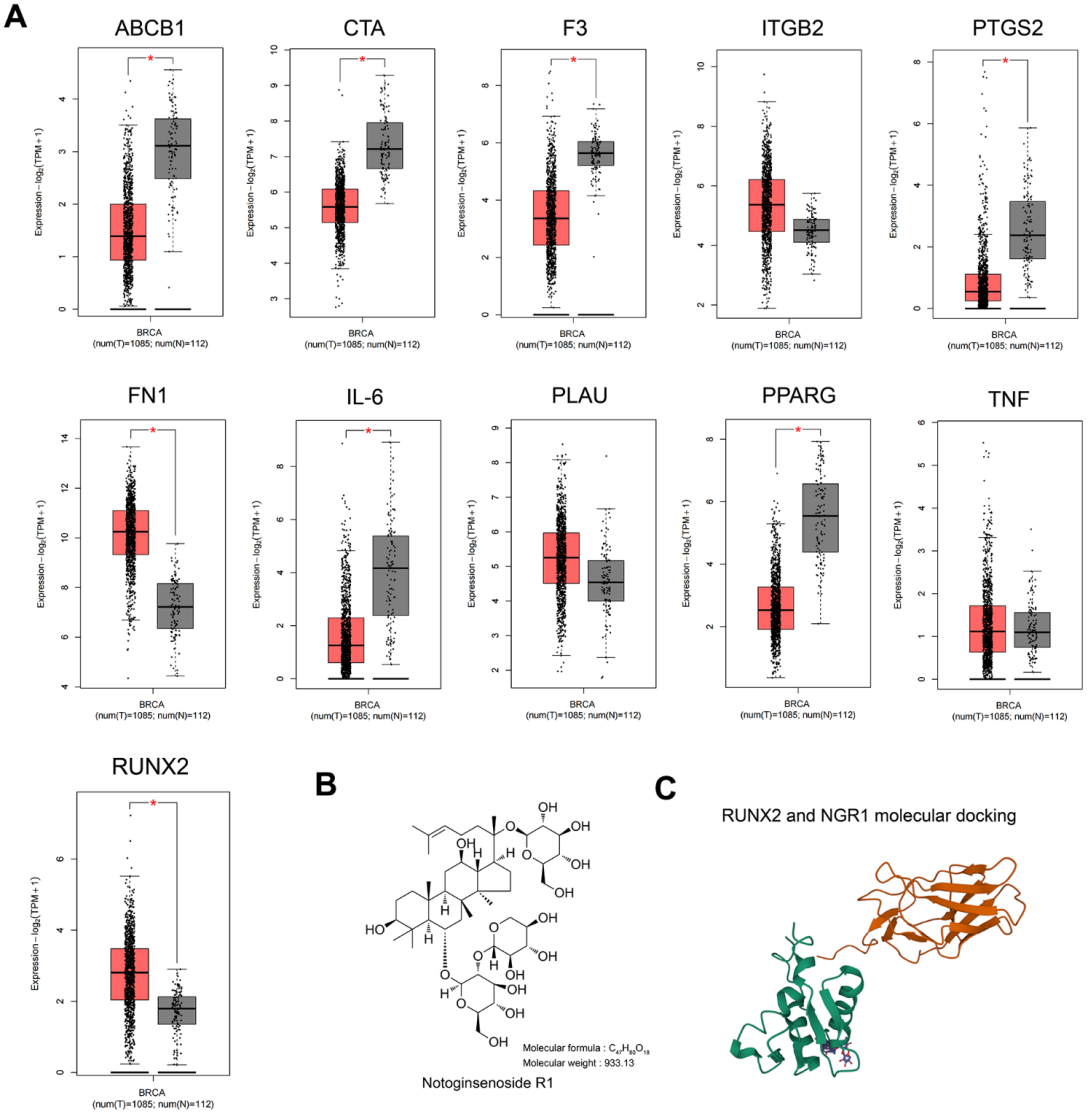
**RUNX2 may be a predictive target of NGR1 in breast cancer.** (**A**) Expression of 11 intersecting genes was examined in the TCGA database of breast cancer patients. (**B**) NGR1 drug structural formula and relative molecular mass. (**C**) NGR1 molecular docking with RUNX2.

ATGC cohort analysis showed that the expression of RUNX2 and FN1 was up-regulated, and the expression of ABCB1, CTA, F3, IL-6 and PPARG was down-regulated in breast cancer tissues compared with that of paraneoplastic tissues, whereas there was no significant difference in the expression of ITGB2, TNF, and PLAU (Figure 2A), and the expression of the differential genes in the GSE205185 and TCGA databases are shown in Supplementary Table 2, the literature search with “NGR1” and “differential gene name” showed that only RUNX2 has a regulatory relationship with NGR1 in osteoblast differentiation [16], and there were no studies on other genes related to NGR1. Therefore, we chose RUNX2 to continue the study in our subsequent experiments. To further demonstrate the interrelationship between NGR1 and RUNX2, our molecular docking results showed that there is a binding site between NGR1 and RUNX2 (Figure 2C).

### NGR1 inhibits RUNX2 expression and slows proliferation of breast cancer cells

**Figure 3:**
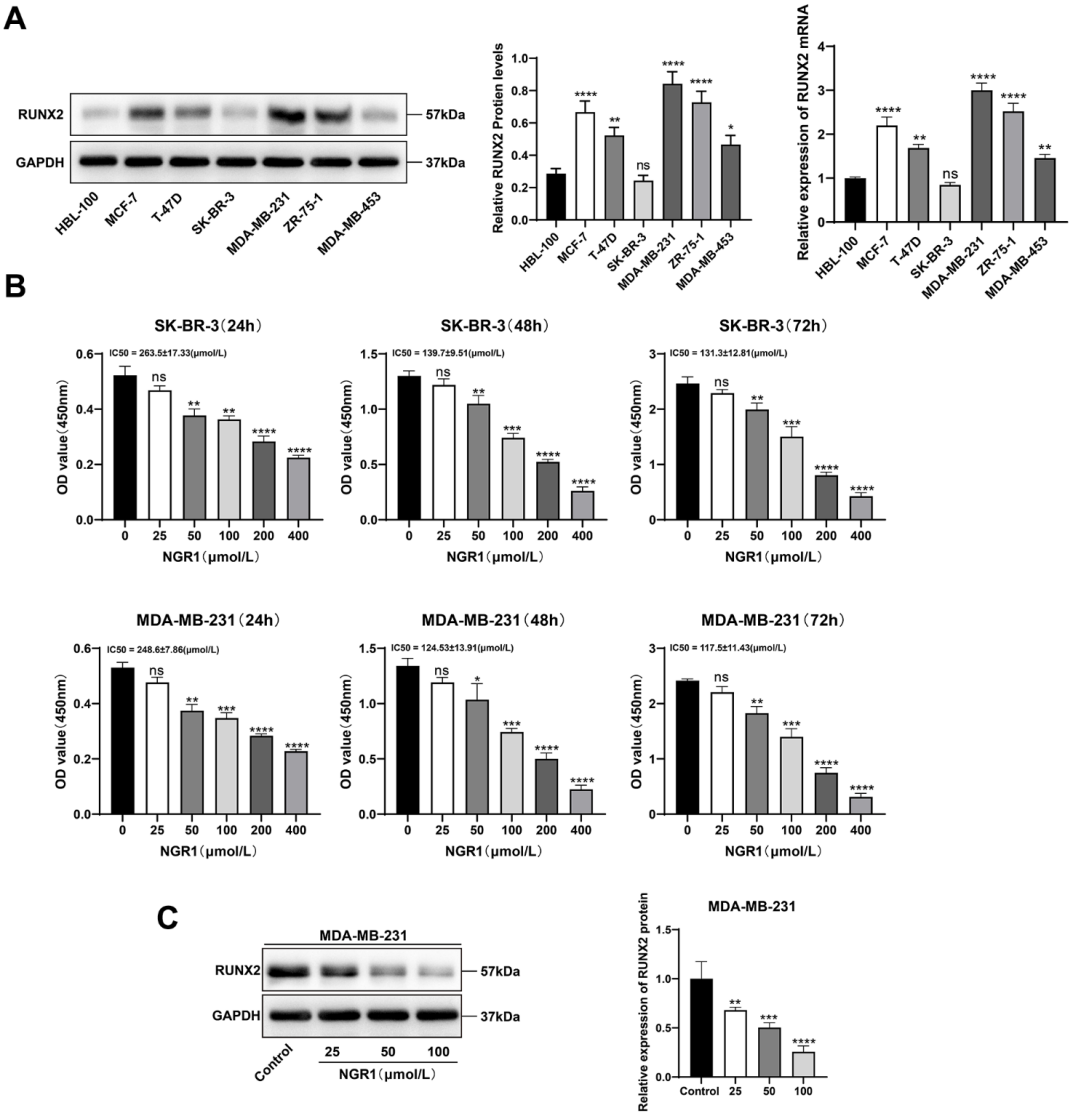
**NGR1 inhibits breast cancer cell viability and RUNX2 expression.** (**A**) The expression levels of RUNX2 protein and mRNA in HBL-100 and different breast cancer cell lines were detected by Western blot assay. (**B**) Changes in cell viability were detected by CCK8 assay after different concentrations of NGR1 (25, 50, 100, 200, 400 μmol/L) were treated with SK-BR-3 and MDA-MB-231 cells for 24 h, 48 h, and 72 h, respectively. (**C**) Western blot detection of RUNX2 protein after treatment of MDA-MB-231 cells with different concentrations of NGR1 (25, 50, 100 μmol/L) for 48 h. (^*^ P < 0.05, ^**^ P < 0.01, ^***^ P < 0.001, ^****^ P < 0.0001).

We examined the mRNA and protein expression levels of RUNX2 in six different breast cancer cell lines and normal cells of mammary epithelium, and the results showed that the mRNA and protein expression levels of RUNX2 were the highest in MDA-MB-231 and the lowest in SK-BR-3 (Figure 3A), and after that, we chose the MDA-MB-231 and SK-BR-3 cells for the follow-up CCK8 results showed that NGR1 was able to inhibit the proliferation of breast cancer cells, and the IC50 of NGR1 on MDA-MB-231 cell line at 24 h, 48 h, and 72 h were 263.5, 139.7, and 131.3 (μmol/L), respectively, and the IC50 of SK-BR-3 on breast cancer cells at 24 h, 48 h, and 72 h were 248.6, 124.53, 117.5 (μmol/L), and the inhibitory effect of NGR1 on breast cancer cells was concentration- and time-dependent (Figure 3B). Subsequently NGR1 was selected at 100 μmol/L for follow-up studies. In addition, NGR1 significantly inhibited the expression level of RUNX2 protein in MDA-MB-231 cells in a concentration-dependent manner (Figure 3C).

**Figure 4:**
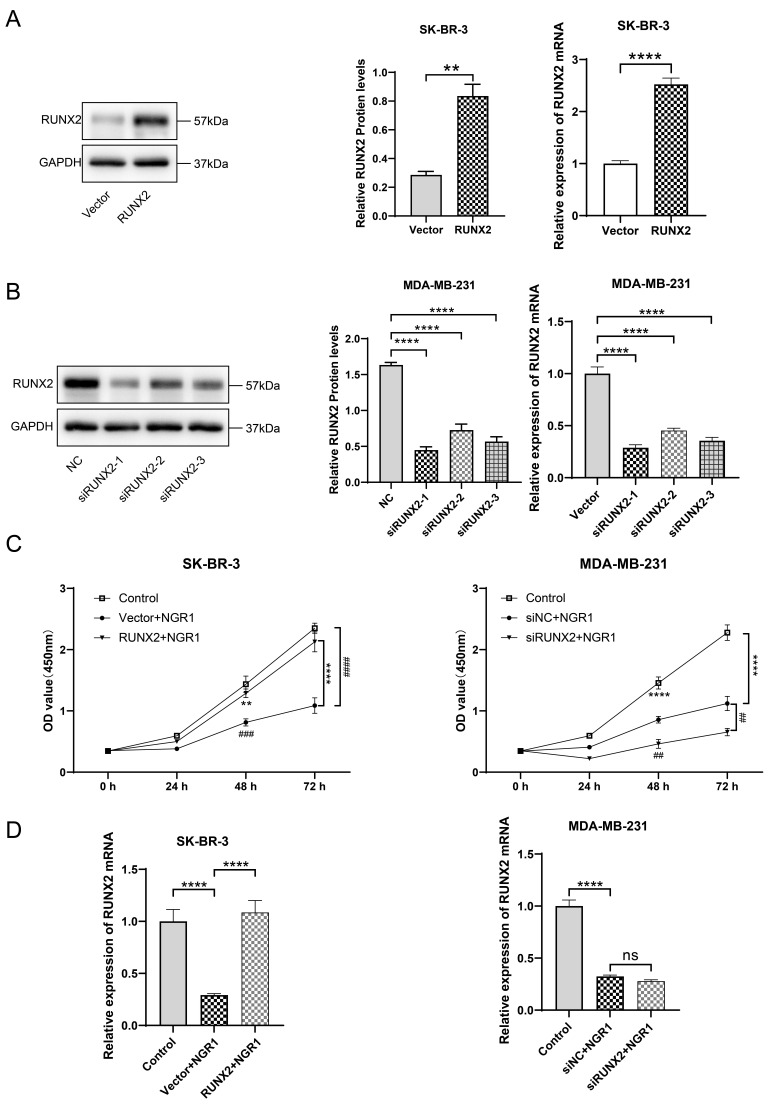
**NGR1 inhibits breast cancer cell proliferation by down-regulating RUNX2 protein expression.** (**A**) After transfection of overexpression RUNX2 plasmid (2 μg/mL) in SK-BR-3 cells for 48 h, the expression of RUNX2 protein and mRNA was detected by Western blot and QPCR assay. (**B**) After transfection of siRNA (100 nmol/L) in MDA-MB-231 cells for 48 h, the expression of RUNX2 protein and mRNA expression. (**C**) Breast cancer cells were transfected with overexpression plasmid and siRNA for 48 h of incubation, followed by NGR1 treatment for 24 h, 48 h, and 72 h. Changes in cell viability were detected by CCK8 assay. (**D**) Expression levels of RUNX2 mRNA were detected by QPCR assay to examine the expression levels of RUNX2 mRNA in breast cancer cell lines. (^*^ P < 0.05, ^**^ P < 0.01, ^***^ P < 0.001, ^****^ P < 0.0001; ^#^ P < 0.0001, ^##^ P < 0.0001, ^###^ P < 0.0001, ^####^ P < 0.0001).

We successfully constructed RUNX2 up-regulated and overexpressed cell lines (Figure 4A, 4B), and overexpression of RUNX2 in SK-BR-3 cells was able to significantly counteract the inhibitory effect of NGR1 on the cells, and the inhibitory effect of NGR1 on the cells was stronger after down-regulation of RUNX2 in MDA-MB-231 cells (Figure 4C). Overexpression of RUNX2 can reverse the inhibitory effect of NGR1 on RUNX2 mRNA expression in SK-BR-3 cells. Transfection with siRUNX2 had no significant effect on RUNX2 mRNA expression in MDA-MB-231 cells treated with NGR1 for 72 h (Figure 4D). This suggests that NGR1 inhibits the proliferation of SK-BR-3 and MDA-MB-231 cells by regulating RUNX2 expression levels.

### NGR1 accelerates ferroptosis in breast cancer cells

**Figure 5:**
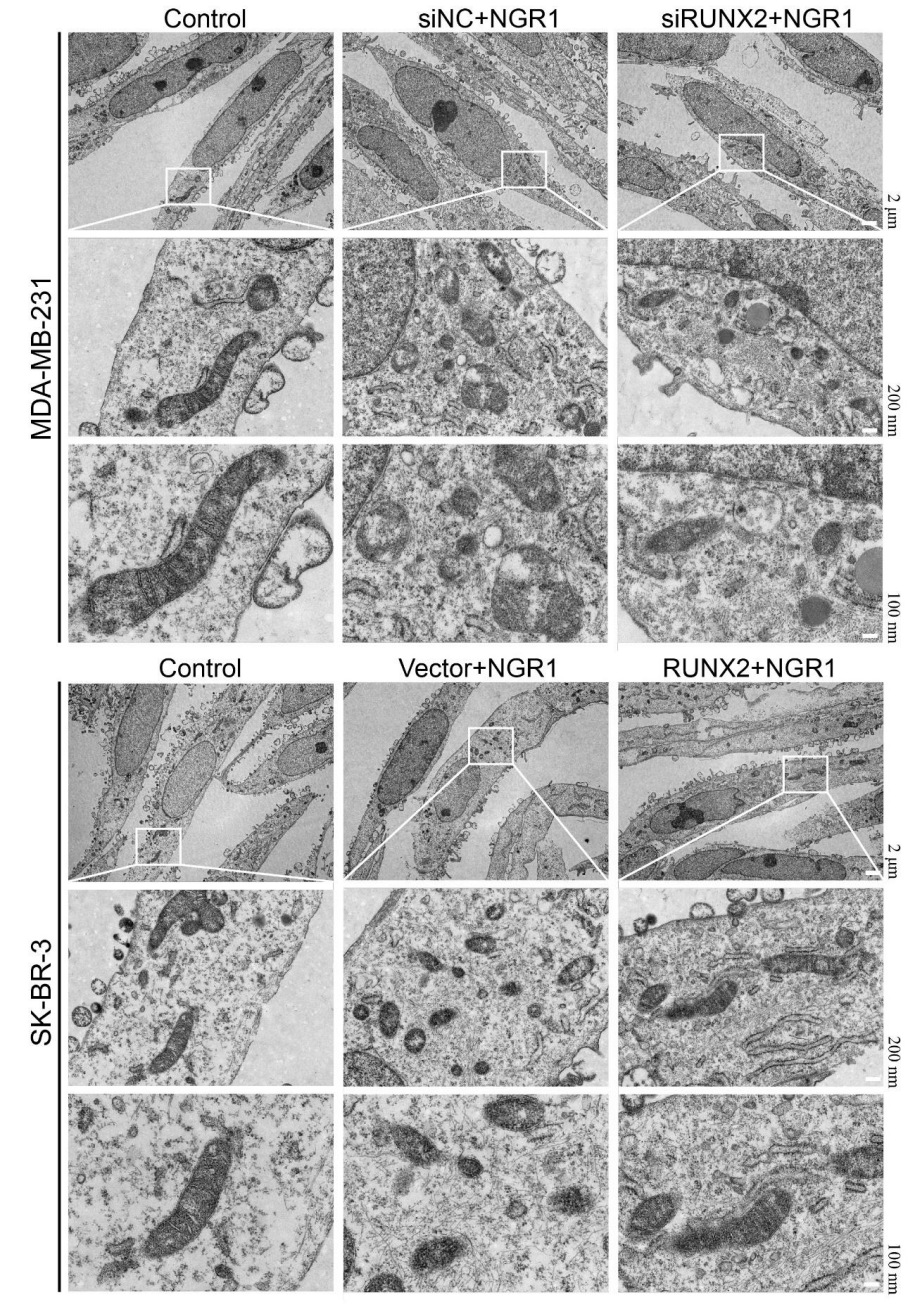
**NGR1 disrupts mitochondrial morphology in breast cancer cells by down-regulating RUNX2.** The mitochondrial structure of breast cancer cells was observed by electron microscopy after transfection of siRNA in breast cancer MDA-MB-231 for 48 h, transfection of overexpression plasmid in SK-BR-3 for 48 h, and then NRG1 treatment for 48 h.

**Figure 6:**
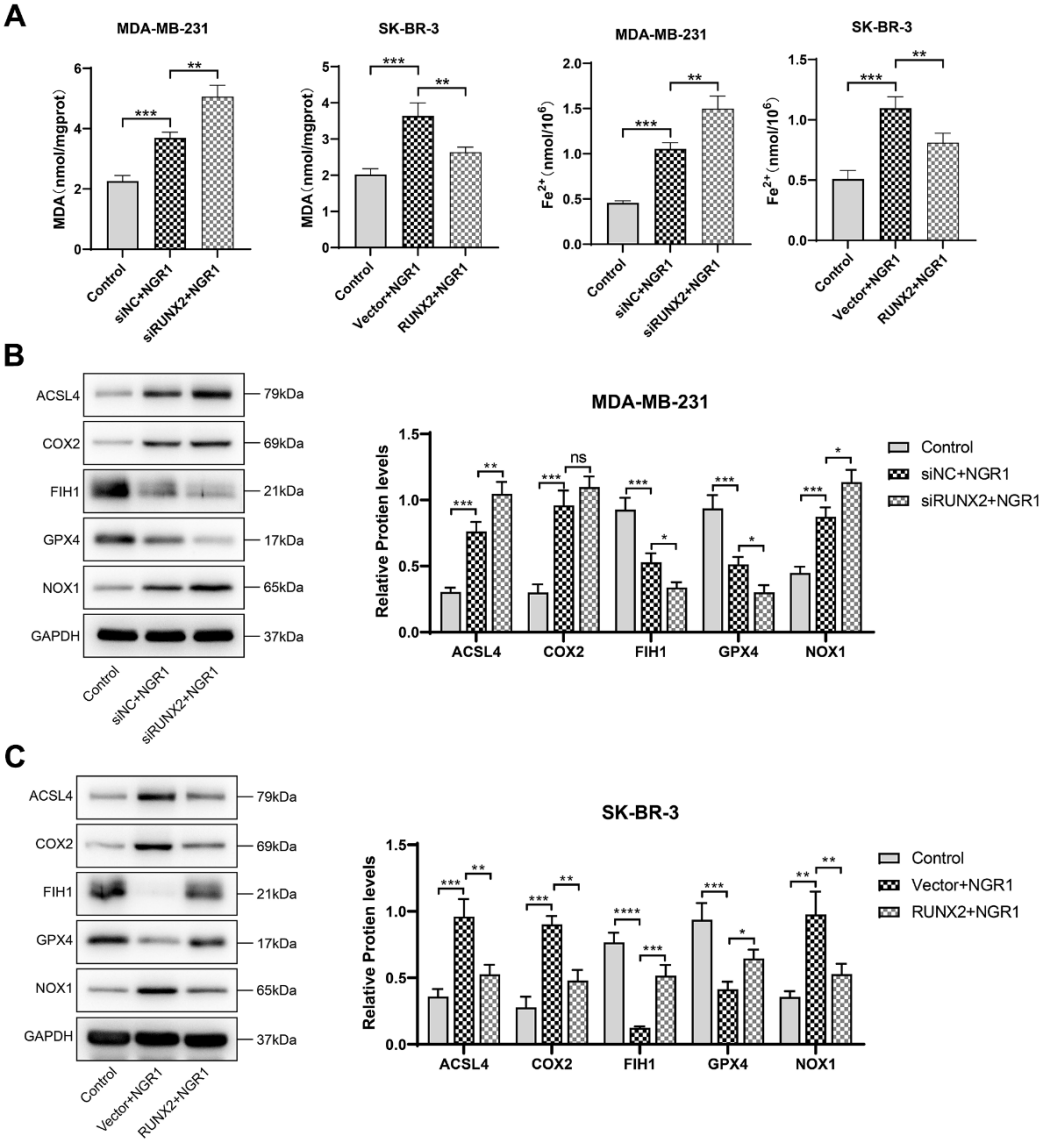
**NGR1 induces ferroptosis in breast cancer cells by down-regulating RUNX2.** (**A**) Changes in the concentration of MDA and Fe^2+^ in MDA-MB-23 and SK-BR-3 cells were detected after NGR1 treatment. (**B**, **C**) Changes in the expression of ACSL4, COX2, FIH1, GPX4, and NOX1 proteins were detected by Western blot in MDA-MB-231 and SK-BR-3 cells. (^*^ P < 0.05, ^**^ P < 0.01, ^***^ P < 0.001, ^****^ P < 0.0001).

We observed by electron microscopy that after NGR1 treatment of breast cancer cells, the mitochondrial membrane was broken, the mitochondria were swollen, the mitochondrial skeleton was disrupted (Figure 5), and the intracellular levels of MDA and Fe^2+^ were significantly increased, and the Western blot results showed that the protein expression levels of ACSL4, COX2, NOX1 ferroptosis inducers were significantly increased after NGR1 treatment. The Western blot results showed that the protein expression levels of ACSL4, COX2 and NOX1 ferroptosis-inducing factors increased significantly after NGR1 treatment, while the protein expression levels of FIH1 and GPX4 ferroptosis inhibiting factors decreased significantly, suggesting that NGR1 was able to induce breast cancer cell ferroptosis. However, the overexpression of RUNX2 could resist the regulation of mitochondrial damage and ferroptosis-related factors by NGR1 in breast cancer cells, and the effect of NGR1 on breast cancer was enhanced after the down-regulation of RUNX2. This demonstrated that NGR1 induced ferroptosis in breast cancer cells by regulating RUNX2 (Figure 6).

### NGR1 inhibits RUNX2 expression and blocks the AGE-RAGE signaling pathway

**Figure 7:**
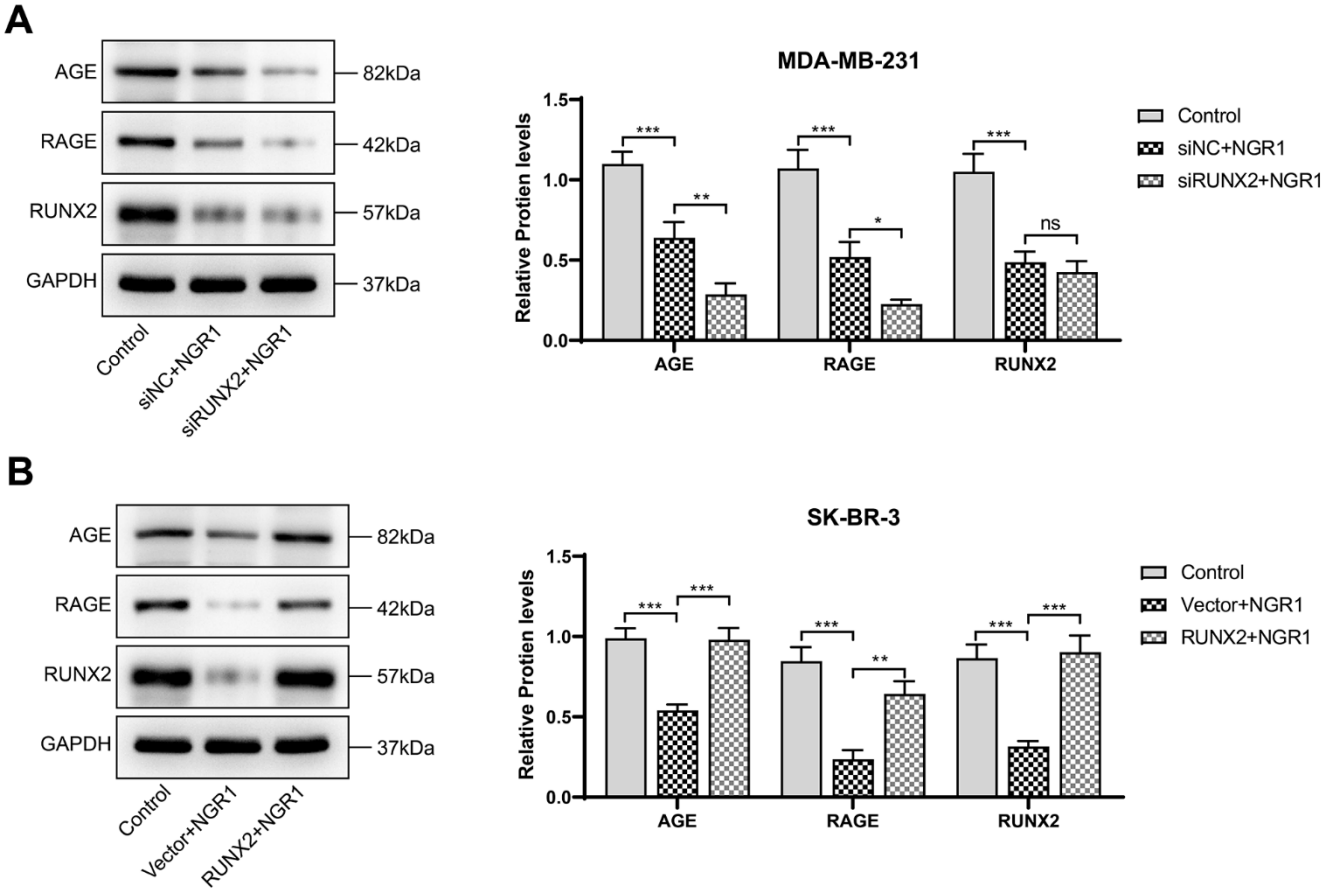
**NGR1 inhibits AGE-RAGE pathway activation by down-regulating RUNX2.** (**A**, **B**) Changes in the expression of AGE, RAGE, and RUNX2 proteins in MDA-MB-231 and SK-BR-3 cells were detected by Western blot assay. (^*^ P < 0.05, ^**^ P < 0.01, ^***^ P < 0.001, ^****^ P < 0.0001).

Western blot results showed that NGR1 could inhibit AGE-RAGE signaling pathway, and after transfecting RUNX2 plasmid and siRNA with overexpression, as expected (Figure 7), NGR1 was likely to inhibit the expression of AGE-RAGE signaling pathway by down-regulating RUNX2.

## DISCUSSION

In recent years, there has been a preliminary understanding of the mechanisms linking breast cancer cells to ferroptosis, but many questions remain for further study. NGR1 has been shown to attenuate breast cancer progression, but its exact mechanism is still unknown [15]. We analyzed a total of 11 genes interacting with NGR1 predicted targets and breast cancer target genes based on online databases, mainly enriched in the AGE-RAGE signaling pathway, which has been shown to correlate with ferroptosis-related factors in periodontitis disease [17], the AGE-RAGE signaling pathway has been shown to be associated with ferroptosis-related factors, and it has been reported in the literature that there is a protein-regulatory relationship with NGR1 and RUNX2 in osteoblast differentiation [16] Our molecular docking results also demonstrated the existence of binding sites for NGR1 and RUNX2. It has been reported in the literature that RUNX2 is able to recruit the NuRD (MTA1)/CRL4B complex and accelerate breast cancer progression and bone metastasis [18] and RUNX2 is also associated with breast cancer drug resistance [19]. Inhibition of RUNX2 expression is a new perspective to prevent breast cancer invasion and metastasis. Our study showed that NGR1 was able to inhibit breast cancer cell proliferation by down-regulating RUNX2 protein and thus, interestingly, we found that MDA-MB-231 was more strongly inhibited by NGR1 compared to SK-BR-3, which may be related to the expression level of RUNX2 in breast cancer cells, which was low in SK-BR-3 cells compared to other breast cancer cells.

Ferroptosis has been reported in an exponentially growing number of studies and shows great potential in a wide range of diseases. Interestingly, tumor-resistant cells and cells undergoing epithelial mesenchymal transition are highly susceptible to ferroptosis [20]. Reports on CRC predicting ferroptosis have indicated that elevated GPX4 expression and decreased NOX1 and FACL4 expression suggest poor prognosis and poor clinical features [21] and protects cells from ferroptosis. In addition, FIH1 and COX2 were able to influence the ferroptosis process by regulating the expression of inflammatory factors [22, 23]. In our study, we found that NGR1 down-regulated RUNX2 to enhance MDA and Fe^2+^ levels and promote oxidative stress and iron ion accumulation in breast cancer cell lines, and NGR1 was also able to activate oxidative stress to inhibit cell growth and invasion in nasopharyngeal carcinoma [24]. NGR1 was also able to up-regulate ACSL4, COX2, NOX1 and down-regulate FIH1, GPX4 protein expression. We also observed that mitochondrial morphology was disrupted in breast cancer cells after NGR1 treatment, and the above results further confirmed that NGR1 was able to induce ferroptosis in breast cancer cells. Subsequently, we focused on the AGE-RAGE signaling pathway based on our previous work, and AGE-RAGE signaling has been validated in the course of cancer and other pathological diseases, and the expression of RAGE increases significantly during cancer progression [25]. Our findings revealed that NGR1 inhibits the AGE-RAGE signaling pathway by down-regulating RUNX2. The reciprocal regulatory relationship between RUNX2 and AGE-RAGE signaling pathways in osteogenic differentiation [26], osteoporosis [27], and smooth muscle cell calcification [28] has also been confirmed by other researchers.

In conclusion, in this paper, we found for the first time that NGR1 can regulate RUNX2 to affect the AGE-ARCE signaling pathway to inhibit the proliferation of breast cells, and our biggest innovation is that we found that NGR1 induces ferroptosis in breast cancer cells, although our results are encouraging, our experiments still have some limitations, and we only carried out cellular experiments *in vitro*, and we have not yet carried out *in vivo* animal experiments to further validate our conclusions. In addition, we are not sure about the regulatory relationship between NGR1 and AGE-ARCE pathway and have not done reverse validation experiments.

## CONCLUSIONS

Our study showed that in breast cancer cells NGR1 inhibits the RUNX2 AGE signaling pathway, induces Fe^2+^ accumulation, enhances intracellular oxidative damage, and thus induces ferroptosis slowing down breast cancer cell proliferation. Therefore, NGR1 could be a potential drug for the treatment of breast cancer, while RUNX2 is a potential target for the treatment of breast cancer, offering the possibility of developing more effective therapeutic strategies.

## MATERIALS AND METHODS

### GSE205185 dataset differential gene volcano and heat maps

The GSE205185 database dataset was downloaded from the GEO database (https://www.ncbi.nlm.nih.gov/gds) and analyzed by principal component analysis (PCA) using the R packages “FactoMineR”, “factoextra”, and “limma”. The R packages “FactoMineR” and “factoextra” were used to perform principal component analysis (PCA) on the two samples, and the differential expression between the two groups was analyzed using the “limma” package. A total of 2346 differentially expressed genes were obtained by screening according to p-value < 0.05&|logFC| > 1. The differential gene volcano map was drawn using the R package “ggplot2”, and the top 20 differentially expressed genes were drawn using the R package “pheatmap”. The R package “ggplot2” was used to draw the differential gene volcano map, and the R package “pheatmap” was used to draw the top 20 differential gene volcano map.

### Wayne plots of NGR1 predicted target genes versus the GSE205185 dataset with PPI network maps

In the CTD database (https://ctdbase.org/), “Notoginsenoside R1” was used as the keyword to download the target information of NGR1 and the differential gene (GSE205185) obtained in the previous section to draw a Venn diagram of the intersecting genes based on the above intersecting gene protein interactions using the R language’s VennDiagram package to draw the intersecting gene Wayne diagram, and the STRING database (https://cn.string-db.org/) was used to construct the protein interactions network based on the above intersecting genes.

### GO and KEGG pathway enrichment analysis

For the 2346 genes analyzed in the GSE205185 dataset, the R package “clusterProfiler” was used to perform GO and KEGG functional enrichment, and the top-ranked results are shown. We obtained 11 intersecting genes from the Wayne diagram, and used the R package “clusterProfiler” to enrich the GO and KEGG functions, and display the results of the top-ranked ones.

### Expression of intersecting genes in other datasets

The GEPIA2 database (http://gepia2.cancer-pku.cn/#index) was used to explore the expression of the above intersecting genes in the TCGA database of breast cancer patients.

### Molecular docking of NGR1 with RUNX2

The molecular structure of NGR1 was obtained from the PubChem Compound Database (https://pubchem.ncbi.nlm.nih.gov/). The 3D coordinates of protein RUNX2 (PDB number: 6VG8; resolution: 4.31 Å) were downloaded from the PDB (http://www.rcsb.org/) and molecular docking was performed using AutodockVina 1.2.2, a computerized protein-ligand docking software.

### Cell culture

Human breast cancer cell lines MDA-MB-231, MCF-7, T-47D, SK-BP-3, MDA-MB-453, ZR-75-1 and human mammary epithelial cell line HBL-100 were purchased from the Shanghai Cell Bank of the Chinese Academy of Sciences. The cells were removed from liquid nitrogen, the cells were resuscitated, and the cells were cultured in DMEM (a mixture containing 10% fetal bovine serum and 1% penicillin streptomycin) medium. NGR1 (HY-N0615) was purchased from MedChemExpress Biotechnology, Inc. (USA).

### Cellular transfection

Breast cancer cell lines in the exponential growth phase were taken, and a premix of Lip2000 (Thermo Fisher Scientific 11668030, USA) with SiRUNX2 interfering sequences or overexpression plasmids was added and incubated in serum-free DMEM medium for 5 h. DMEM medium was supplemented and continued to be incubated for 48 h for the subsequent experiments. The relationship of SiRNA and plasmid sequences are detailed in Supplementary Table 1. The map of the overexpression plasmid is shown in detail in Supplementary Figure 1.

### Cell counting kit-8 assay

Cells were transfected as previously described, and the successfully transfected cells were taken and inoculated in 96-well plates at a density of 3000/cells for culture, and incubated with different concentrations of NGR1 for 0 h, 24 h, 48 h, and 72 h, respectively, and then 10 μL of CCK8 solution (Beyotime Biotechnology, C0038, China) was added to each well to continue the incubation for 4 h. The OD (450 nm) values of the 96-well plates were taken out and placed on SpectraMax Mini Multifunctional Enzyme Labeler.

### QPCR assay

Take the successfully transfected cells, add appropriate amount of Trizol solution, extract the total RNA from the cells according to the instructions, determine the concentration of RNA by using NanoDrop One, reverse transcribe the cDNA according to the requirements of BeyoRT™ II cDNA First Strand Synthesis Kit (Beyotime Biotechnology, D7168M, China), and add BeyoFast™ SYBR Green qPCR Mix (Beyotime Biotechnology, D7262, China) premix solution. BeyoFast™ SYBR Green qPCR Mix (Beyotime Biotechnology, D7262, China) was added as a premixing solution, and the ABI 7900HT Fluorescence PCR instrument was set up according to the instruction manual, and the results were analyzed using the software provided with the Fluorescence PCR instrument after the operation was completed.

### Measurement of Fe^2+^ content with MDA detection

The transfected cells were taken, and the cell precipitates were extracted by expanding each group of cells to more than 2×10^6^ and set aside. Cell samples were processed according to the requirements of the iron content detection kit (Merck, MAK025, Germany) to detect the Fe^2+^ content in the cells, and the OD (593 nm) value was detected using a SpectraMax Mini multifunctional enzyme marker. Cells were treated according to the requirements of the MDA assay kit (Beyotime Biotechnology, S0131S, China), and the OD (532 nm) value was detected using a SpectraMax Mini multifunctional enzyme marker.

### Changes in mitochondrial morphology observed by electron microscopy

The transfected cells were removed, collected with a spatula, and incubated for 4 h with electron microscope fixation (Servicebio, G1102, China) solution, 1% agarose was added to dissolve the agarose, the sample wrapped in agarose was taken out and incubated with 1% osmium (Ted Pella Inc., 18456, USA) for 2 h at room temperature away from light, and the tissues were dehydrated by a gradient of alcohol, and then added to the embedding agent (SPI, 90529-77-4) and incubated overnight in an oven at 37° C. Ultrathin sections were then sliced with copper mesh. The tissue was incubated in a 37° C oven overnight, and the embedded plates were incubated in a 60° C oven for 48 h. After ultrathin sectioning, the slices were fished with a copper mesh, which was incubated in a 2% uranyl acetate saturated alcoholic solution for 8 min in the light, and then cleaned with alcohol and water, and then incubated with a 2.6% lead citrate solution for 8 min, and then dried overnight and the images were visualized by a transmission electron microscope (Hitachi, HT7800, Japan).

### Western blot assay

Take the successfully transfected cells, add the tissue cell lysate to incubate the cell samples for 30 min, extract the cell protein solution, determine the protein concentration by BCA kit, adjust the amount of sample according to the protein concentration, add an equal amount of protein solution to the groove of the gel, set the voltage at 120 V to perform gel electrophoresis, transfer the gel to the PVDF membrane after 75 min, after that, the conditioned voltage was adjusted to 200V, after 30 min, the PVDF membrane was put into 5% skimmed milk powder and incubated for 2 h. After the incubation, the membrane was washed three times by TBST, the primary antibody was washed three times by TBST after overnight incubation, and the secondary antibody was incubated for 2 h. After the incubation, chemiluminescent solution was added and the membrane was incubated for 10 s, and then put into chemiluminescent instrument to preserve the images for subsequent statistical analysis. GPX 4 (#DF6701), FIH1 (#DF7354), COX2 (#AF7003), ACSL4 (#DF12141), NOX1 (#DF8684), GAPDH (#AF7021), Goat Anti-Rabbit IgG (H+L) HRP (#S0001), Goat Anti-Mouse IgG (H+L) HRP (#S0002) were purchased from Affinity Biosciences, USA. PTGS2 (ab255420), AGE (ab23722), RAGE (ab216329) were purchased from Abcam, UK.

### Statistical analysis

GraphPad Prism 9.5.0 software was adopted for data analysis. Measurement data are expressed as mean ± standard deviation (SD). Paired samples t-tests assessed the significance of 11 differential genes in breast and adjacent cancer expression. Western blot and CCK8 data results were evaluated by two-way analysis of variance (ANOVA), and other data were evaluated using one-way ANOVA, followed by the post hoc comparisons with Tukey’s honestly significant difference test. P<0.05 indicates the difference is statistically significant.

### Availability of data and materials

The datasets used and/or analyzed during the current study are available from the corresponding author on reasonable request.

## Supplementary Material

Supplementary Figure 1

Supplementary Tables
