# Supplementary figures and images for: Notoginsenoside R1 (NGR1) regulates the AGE-RAGE signaling pathway by inhibiting RUNX2 expression to accelerate ferroptosis in breast cancer cells
Source: Aging (Albany NY). 2024 Jun 14;16(12):10446–61. doi: 10.18632/aging.205940 (PMC11236304; doi:10.18632/aging.205940)

SUPPLEMENTARY FIGURE

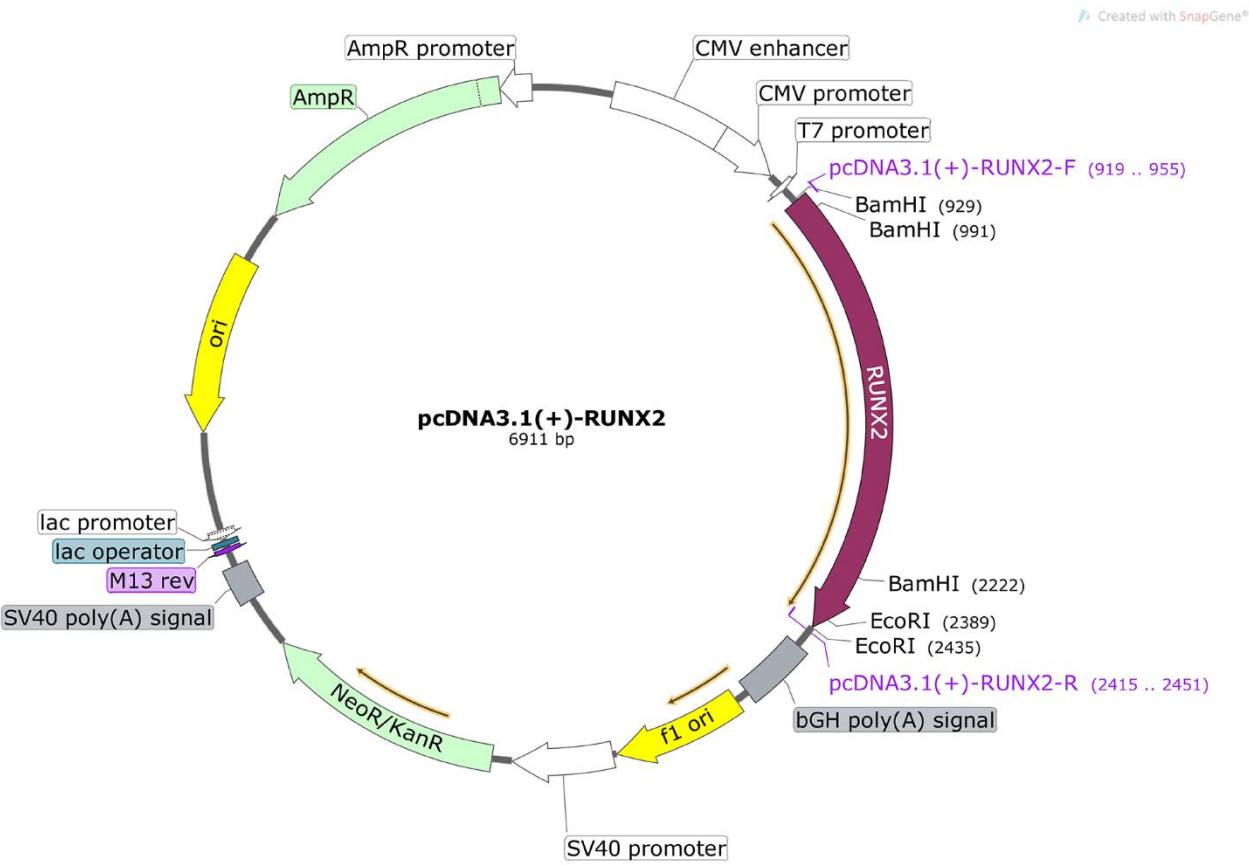

Supplementary Figure 1. RUNX2 overexpression plasmid construction map.

Supplement: Supplementary Figure 1 [file aging-16-205940-s001.pdf]
