# Supplementary material for: Notoginsenoside R1 (NGR1) regulates the AGE-RAGE signaling pathway by inhibiting RUNX2 expression to accelerate ferroptosis in breast cancer cells
Source: Aging (Albany NY). 2024 Jun 14;16(12):10446–61. doi: 10.18632/aging.205940 (PMC11236304; doi:10.18632/aging.205940)
Supplement: Supplementary Tables [file aging-16-205940-s002.pdf]

SUPPLEMENTARY TABLES

Supplementary Table 1. QPCR primer sequences and the siRNA interference sequences and the MYLK overexpression sequences.

Gene	Forward primer (5′–3′)	Reverse primer (5′–3′)
RUNX2	CTACCAGTGTTTGGAGGGCA	ATGCGTATGTGTGTATCCGCCC
GAPDH	GATTCCACCCATGGCAAATTC	CTGGAAGATGGTGATGGGATT
NC	GGAAGUCUGUUAUCAGAUAUC	UAUCUGAUAACAGACUUCCUG
Gene	SS Sequence	AS Sequence
SiNC	GGUCUGUUAUAAUCAAUAACC	UUAUUGAUGAAAACAGACCAA
SiRUNX2-1	GGUCUGUUAUCAUCAAUAACC	UUAUUGAUGAUAACAGACCAA
SiRUNX2 -2	GCAAAGACAUAGUCAGCUAAA	UAGCUGACUAUGUCUUUGCUA
SiRUNX2 -3	GUGUUGAAAUGUUACUAUAGU	UAUAGUAACAUUUCAACACAA
Gene	Primer sequence (5 ‘-3’)	
pcDNA3.1-MYLK-F	TACCGAGCTCGGATCCATGGGGGATGTGAAGCTGGTTGC	
pcDNA3.1-MYLK-R	GATATCTGCAGAATTCTCACTCTTCTTCCTCTTCCCCTTCCCCTTCAC	

Supplementary Table 2. Summary of intersection gene expression in different data.

gene	In this analysis, GSE205185 gene expression was used	Gene expression in TCGA breast cancer
PPARG	Down	Down
PTGS2	Down	Down
F3	Down	Down
IL6	Down	Down
CAT	Down	Down
ABCB1	Down	Down
ITGB2	Up	-
FN1	Up	Up
TNF	Up	-
RUNX2	Up	Up
PLAU	Up	-
